# Supplementary material for: Investigating the Product Profiles and Structural Relationships of New Levansucrases with Conventional and Non-Conventional Substrates
Source: Int J Mol Sci. 2020 Jul 29;21(15):5402. doi: 10.3390/ijms21155402 (PMC7432509; doi:10.3390/ijms21155402)
Supplement: Supplementary file 1 [file ijms-21-05402-s001.pdf]

Supplemental Material

Table S1: Interactions between enzyme residues and carbohydrate

		Interactions		
**Substrate**	**Enzyme**	**Substrate Contacts**	**H-Bonds (Donor Sugar)**	**H-Bonds (Donor Enzyme)**
Sucrose	*B. subtilis*	D86, S164, L109, Y429, R343, D247, Y411, R360, R246, W163	Y429, D86	S164
Sucrose	*B. amyloliquefaciens*	R213, E307, R327, R400, E309	E307, E309	R327
Sucrose	*B. indica*	D98, F421, T361, R445 P409 H359 Y410, D439, D371, H101	D439, D371, D98	H101, R445
Sucrose	*G. oxydans*	H80, T365, D313, W79, R82, W346, D364, H301	D364, D313	R82, W79
Sucrose	*P. graminis*	R101, R434, H102, P398, P350, D99, Y399, H348	D99	R101
Sucrose	*V. natriegens*	R234, H345, Y230, E327, N232, Q325	E327, Q325, N232	H345, Q325
Fructose	*B. subtilis*	D247, Y411, S412, A342, D86, R246, E262, R343	D86, E262, D247	S412, R246
Fructose	*B. amyloliquefaciens*	R213, E307, R327, R400, E309	E309, D53	Y52
Fructose	*B. indica*	F244, S246, R248, D338, Q339	ASP338, GLN339	ARG248
Fructose	*G. oxydans*	S120, W41, N83, R194, W119, D195, E283	D195, E283	R194, N83, S120
Fructose	*P. graminis*	H102, D99, R434, F98, D428, R101	D99	H102, R101
Fructose	*V. natriegens*	R175, Y230, G179, N180, D181	D181	N180, Y230, D181, R175
Glucose	*B. subtilis*	D247, S164 D86, R246, L109, W163	D247, D86, S164	-
Glucose	*B. amyloliquefaciens*	W130, E309, R213, D214, L76, W52, S131	-	W52, S131
Glucose	*B. indica*	D98, R100, H101, P409, Y410, D439, R445	D98, R100, R445	H101
Glucose	*G. oxydans*	S120, R194, L65, W41, D195, W119	D195	S120, R194
Glucose	*P. graminis*	R434, H102, R101, F98, D428, D99	D99	H102, R101
Glucose	*V. natriegens*	R435, H100, D425, D97, P395, R99	D425, D97	R99

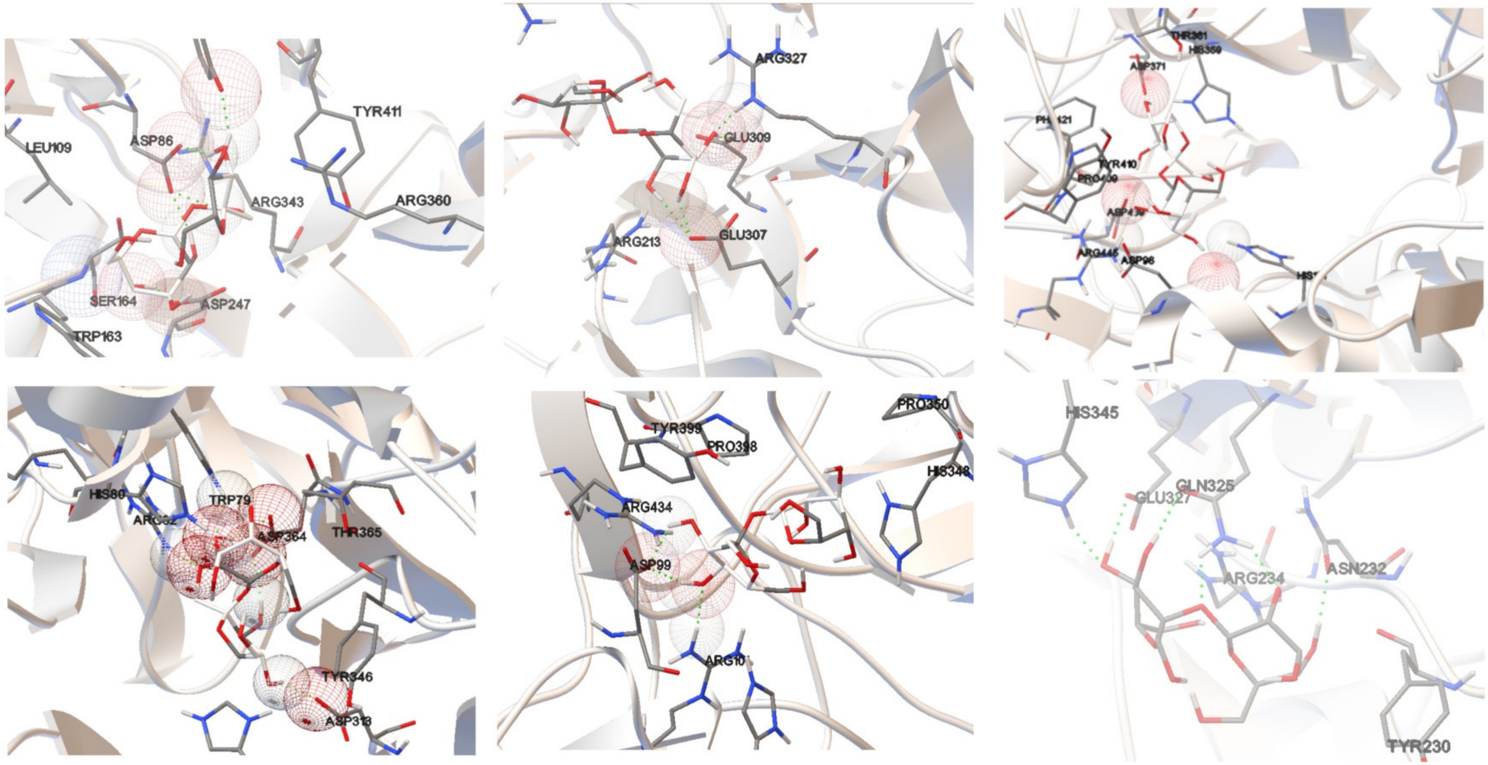

Figure S1: Models of LS from a) B. subtilis, (b) B. amyloliquefaciens, (c) B. indica subsp. indica, (d) G. oxydans, (e) P. graminis and *(f) V. natriegens with sucrose docked within the active site. Sucrose is docked in a position representing the least binding energy state. Colouring represents electrostatic surface potential. The surface charge was calcuated using AutoDock v 4.2 and the PDBqt file was uploaded to OPAL webserver for PQR map and visualization was done using PyMol APBS plugin.*

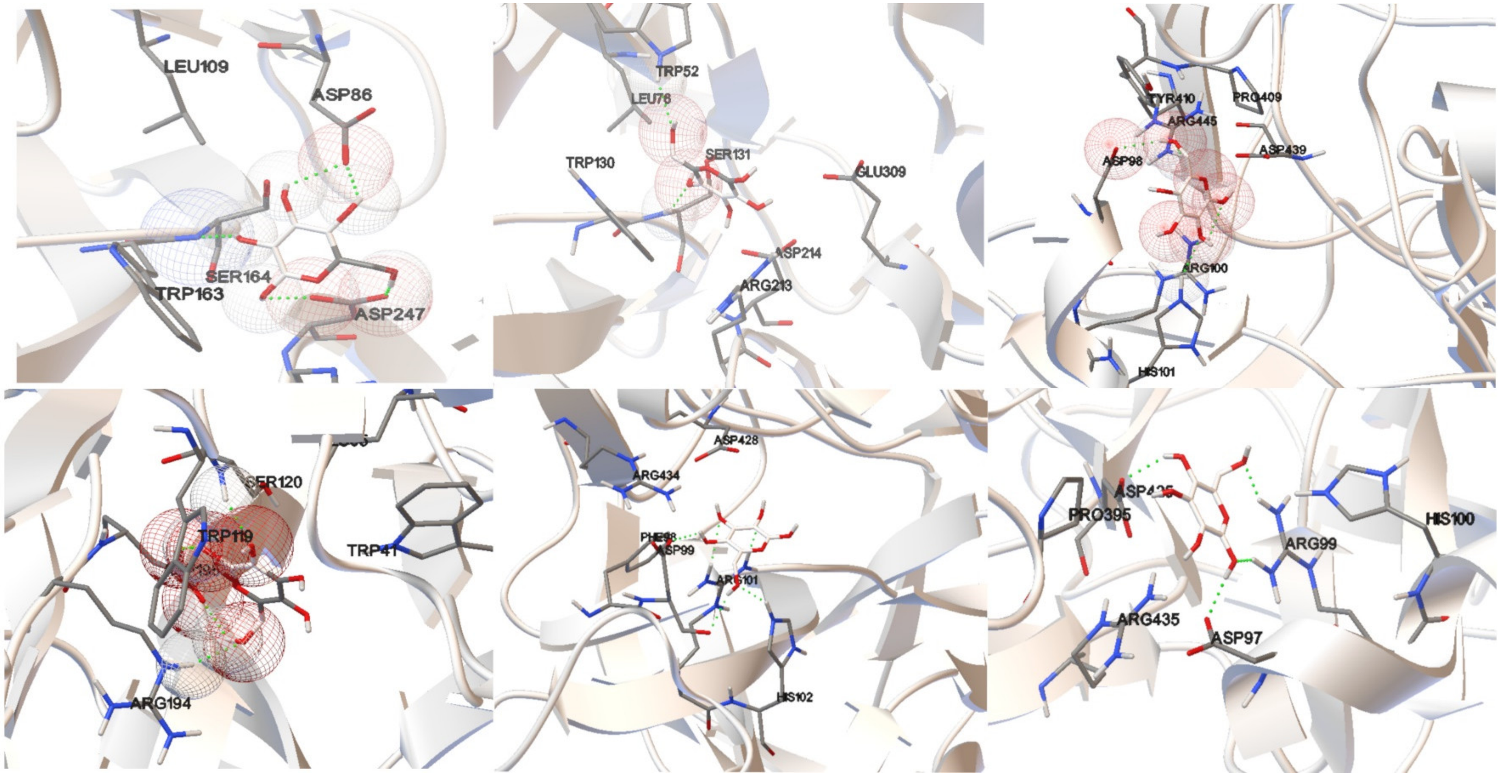

Figure S2: Models of LS from a) *B. subtilis*, *(b) B. amyloliquefaciens, (c) B. indica subsp. indica*, *(d) G. oxydans*, *(e) P. graminis* and *(f) V. natriegens with glucose docked within the active site. Glucose is docked in a position representing the least binding energy state. Colouring represents electrostatic surface potential. The surface charge was calculated using AutoDock v 4.2 and the PDBqt file was uploaded to OPAL webserver for PQR map and visualization was done using PyMol APBS plugin.*

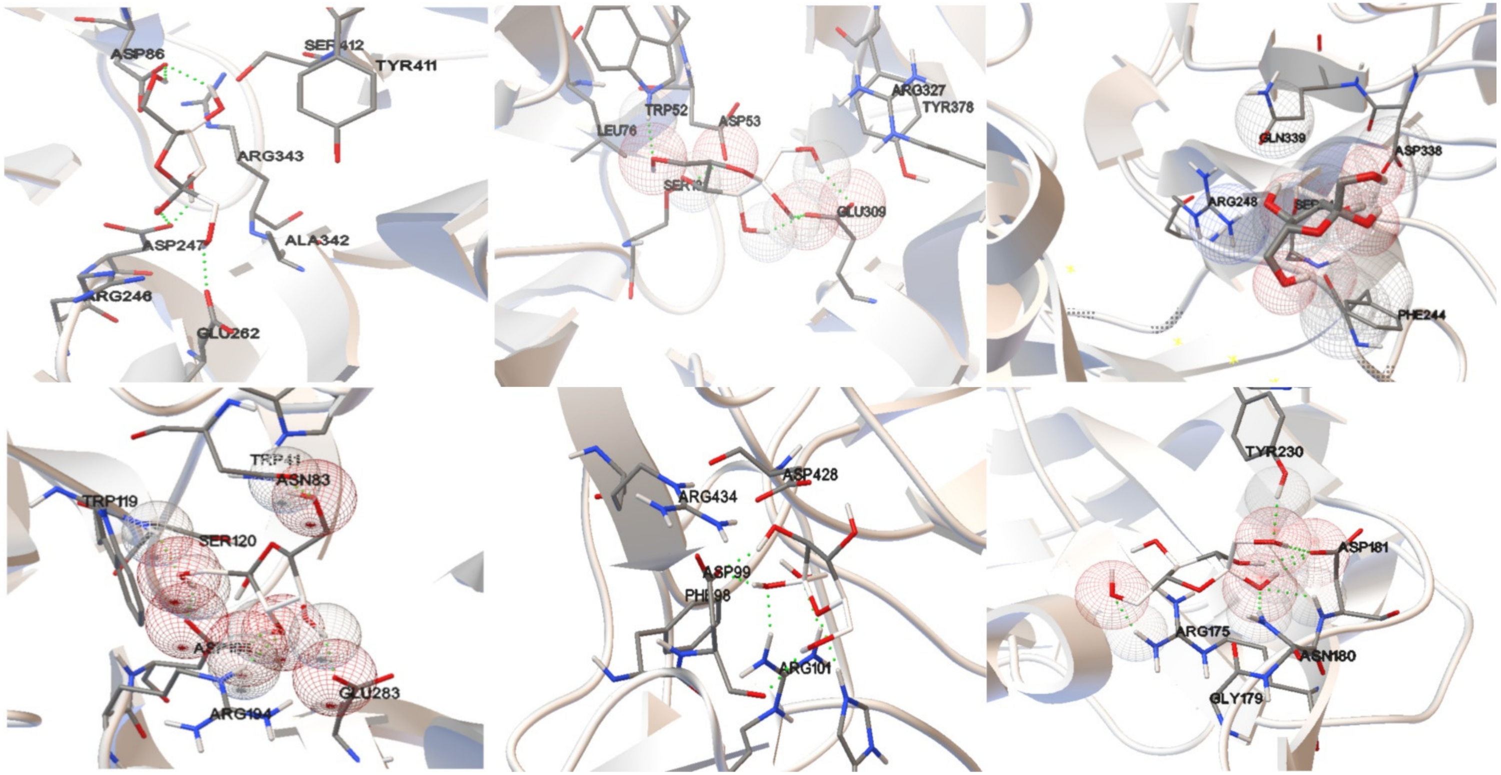

Figure S3: Models of LS from a) *B. subtilis*, *(b) B. amyloliquefaciens, (c) B. indica subsp. indica*, *(d) G. oxydans*, *(e) P. graminis* and *(f) V. natriegens with fructose docked within the active site. Fructose is docked in a position representing the least binding energy state. Colouring represents electrostatic surface potential. The surface charge was calculated using AutoDock v 4.2 and the PDBqt file was uploaded to OPAL webserver for PQR map and visualization was done using PyMol APBS plugin.*

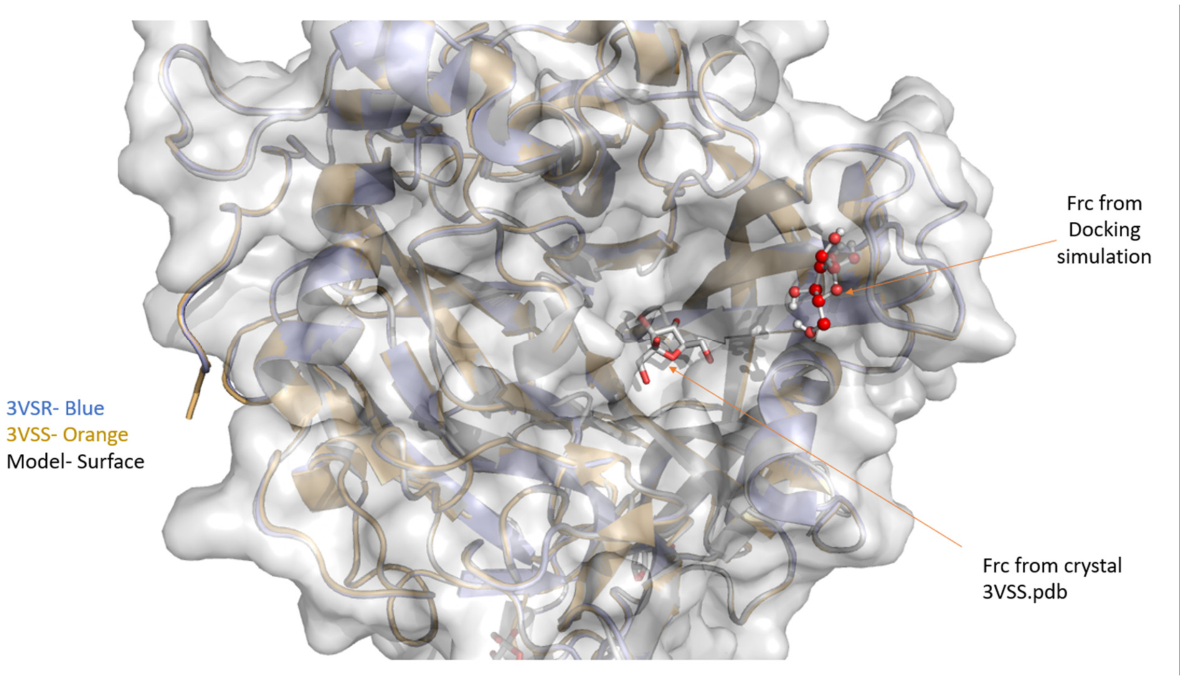

Figure S4: Comparison of the fructose docked in a position representing the least binding energy state generated by docking simulations using the unliganed (3VSR) LS homology model for V. natriegens. It was developed using Microbacterium saccharophilum K-1 beta-fructofuranosidase. The image shows 3VSR (unliganged), 3VSS (bound Frc).

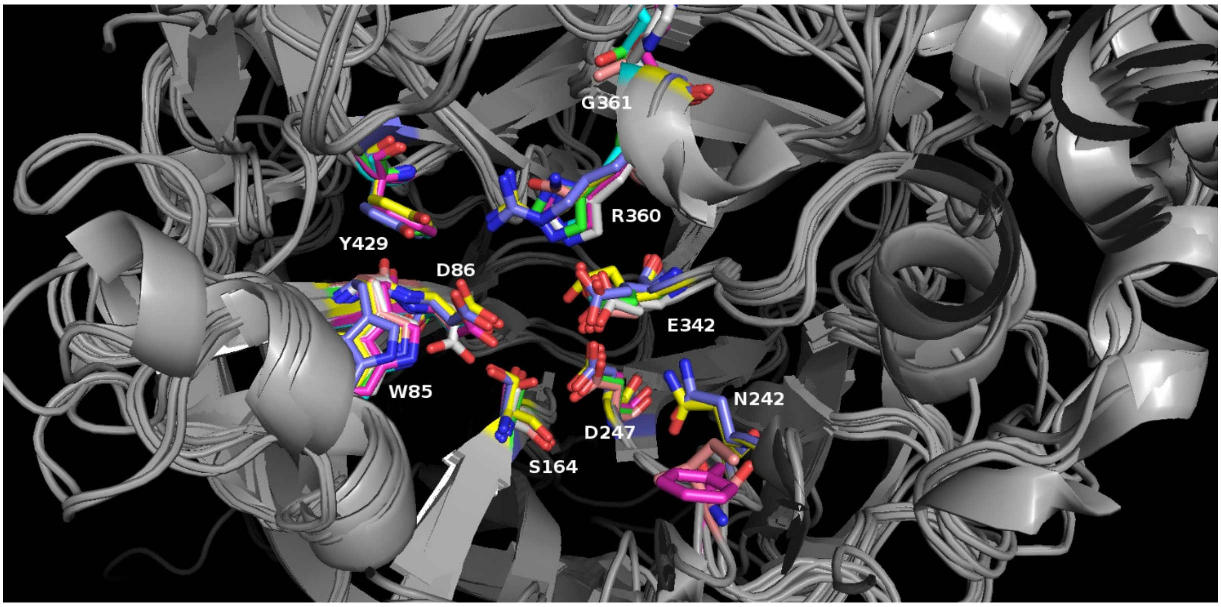

Figure S5: Overlay of the homology models developed for the LSs in this study.

Table S2: Percent bioconversion of various monosaccharides, disaccharides, and trisaccharides as acceptor/donor molecules by various LSs as a function of time

		Vibrio natriegens	*Gluconobacter oxydans*	*Novosphingobium aromaticivorans*	*Burkholderia graminis*	*Berjinckia indica subsp. indica*
	Reaction time (h)	Average conversion (%)	Average conversion (%)	Average conversion (%)	Average conversion (%)	Average conversion (%)
0.9 M Sucrose	2	34.1 ± 11.9	33.9 ± 3.4	20.5 ± 7.3	16.9 ± 0.2	23.4 ± 0.7
	24	54.7 ± 6.4	61.5 ± 3.3	23.0 ± 6.0	61.0 ± 1.4	51.0 ± 1.0
	50	59.7 ± 6.1	77.9 ± 0.5	52.9 ± 3.8	62.5 ± 6.7	72.2 ± 2.1
0.9 M Raffinose	2	37.1 ± 2.9	92.7 ± 0.8	32.4 ±	54.9 ± 20.1	80.9 ±
	24	61.1 ± 2.1	48.4 ±	64.6 ± 6.0	30.4 ±	-
	50	73.0 ± 2.7	58.3 ± 5.4	64.0 ± 2.9	68.2 ± 5.5	66.5 ± 7.8
0.45 M Raffinose / 0.9 M Sucrose	2	52.2 ± 14.1	27.2 ±	26.4 ± 3.	11.7 ± 4.9	7.7 ±
	24	52.2 ± 3.2	20.5 ± 5.0	27.6 ± 2.6	36.7 ±	15.9 ±
	50	69.7 ± 12.4	71.0 ± 6.0	56.9 ± 2.8	69.7 ± 3.4	66.3 ± 7.9
0.45 M Galactose / 0.9 M Sucrose	2	73.3 ± 4.9	52.3 ± 5.7	12.9 ±	48.0 ± 3.0	58.8 ± 1.8
	24	76.2 ± 1.2	50.4 ± 3.1	41.7 ± 19.7	48.9 ± 7.3	66.6 ± 0.3
	50	34.2 ± 2.9	45.9 ± 2.4	46.9 ± 0.7	58.2 ± 2.5	46.2 ± 4.3
0.45 M Maltose / 0.9 M Sucrose	2	38.3 ± 15.3	34.4 ±	55.2 ±	59.8 ±	62.2 ± 0.2
	24	38.0 ±	41.7 ± 11.9	68.8 ± 3.2	58.6 ±	73.8 ± 0.1
	50	72.0 ± 5.4	52.5 ± 6.4	38.6 ± 0.2	56.1 ± 11.3	51.3 ± 1.1
0.45 M Lactose / 0.9 M Sucrose	2	68.7 ± 4.2	46.2 ± 8.6	67.9 ± 12.0	53.07 ± .02	54.9 ± 3.2
	24	67.1 ± 28.4	65.8 ± 8.2	18.9 ± 13.2	53.5 ± 7.5	78.1 ± 1.5
	50	60.4 ± 17.0	44.1 ± 1.8	67.2 ± 4.0	78.7 ± 2.6	71.1 ± 1.0
0.45 M Xylose / 0.9 M Sucrose	2	84.3 ± 1.8	60.6 ± 4.0	84.0 ± 4.0	62.1 ± 11.4	60.2 ± 13.4
	24	79.3 ±	66.8 ± 7.1	73.6 ± 2.0	62.3 ± 10.2	73.3 ± 2.4
	50	70.0 ± 8.5	62.8 ± 6.1	73.1 ± 4.3	67.2 ± 5.0	73.1 ± 0.3
0.45 M Sorbitol / 0.9 M Sucrose	2	59.3 ± 2.8	39.0 ± 25.0	29.8 ± 17.2	25.9 ± 4.3	49.6 ± 3.9
	24	59.4 ± 1.3	45.0 ± 0.9	43.4 ±	-	55.7 ± 1.2
	50	50.1 ± 2.0	45.4 ± 5.1	43.8 ± 2.4	42.0 ± 6.1	36.1 ± 2.8
0.45 M Catechol / 0.9 M Sucrose	2	-	6.8 ±	29.8 ± 17.2	-	50.3 ± 4.1
	24	20.1 ±	-	17.3 ±	-	-
	50	45.2 ± 16.5	-	38.8 ± 0.6	36.4 ± 18.3	61.1 ± 5.1
